# Plant *In Vitro* Production of Phenolic Bioactives: Molecular Regulation, Functional Equivalence and Translational Challenges

**DOI:** 10.3390/ijms27156996

**Published:** 2026-08-04

**Authors:** Anna Kujawska, Paulina Król, Oleksandra Laban, Piotr Karczyński

**Affiliations:** Institute of Biology, University of Szczecin, Wąska 13, 71-415 Szczecin, Poland; paulina.krol@usz.edu.pl (P.K.); oleksandra.laban@usz.edu.pl (O.L.); piotr.karczynski@usz.edu.pl (P.K.)

**Keywords:** plant *in vitro* cultures, phenolic compounds, phenolic bioactives, molecular regulation, functional equivalence, translational applications

## Abstract

Phenolic compounds are important plant secondary metabolites with broad biological activity and potential applications in pharmaceutical, food, cosmetic, nutraceutical, and veterinary sectors. Conventional production from field-grown plants is limited by environmental variability, seasonality, and difficulties in standardizing metabolite composition. Plant *in vitro* cultures provide controlled systems for modulating secondary metabolism and producing phenolic compounds under defined conditions. This review summarizes current advances in plant *in vitro* platforms for phenolic production and discusses regulation through elicitation, metabolic modulation, molecular approaches, and bioreactor cultivation. The distinctive focus of this review is the critical evaluation of how culture type, production stability, metabolite composition, and structural variation affect biological performance and functional equivalence. Current evidence indicates that increased metabolite accumulation alone does not ensure preserved biological properties or translational applicability. Functional equivalence is therefore considered as a framework integrating chemical profiling, batch-to-batch reproducibility, biological validation, bioavailability, and application-oriented evaluation of *in vitro*-derived phenolics. Future progress will depend not only on increasing yield but also on achieving stable production, predictable composition, reproducible biological performance, and translational reliability.

## 1. Introduction

Phenolic compounds constitute one of the largest groups of plant secondary metabolites. In this review, the term phenolic bioactives refers to individual phenolic compounds or phenolic-rich preparations that show measurable activity in a defined experimental model. This term does not imply therapeutic efficacy or clinical benefit. Synthesized mainly through the shikimate and phenylpropanoid pathways, they participate in numerous physiological processes associated with plant growth, development, and responses to environmental conditions. Phenolics contribute to antioxidant defence, protection against UV radiation, pathogen resistance, signalling, and maintenance of structural integrity in plant tissues. Owing to their structural diversity and broad spectrum of biological activity, phenolic compounds have attracted considerable interest because of their potential applications in pharmaceutical, food, cosmetic, nutraceutical, and veterinary sectors. Numerous studies have demonstrated antioxidant, anti-inflammatory, antimicrobial, cardioprotective, neuroprotective, and metabolic effects of phenolic compounds [1,2,3,4].

**Figure 1:**
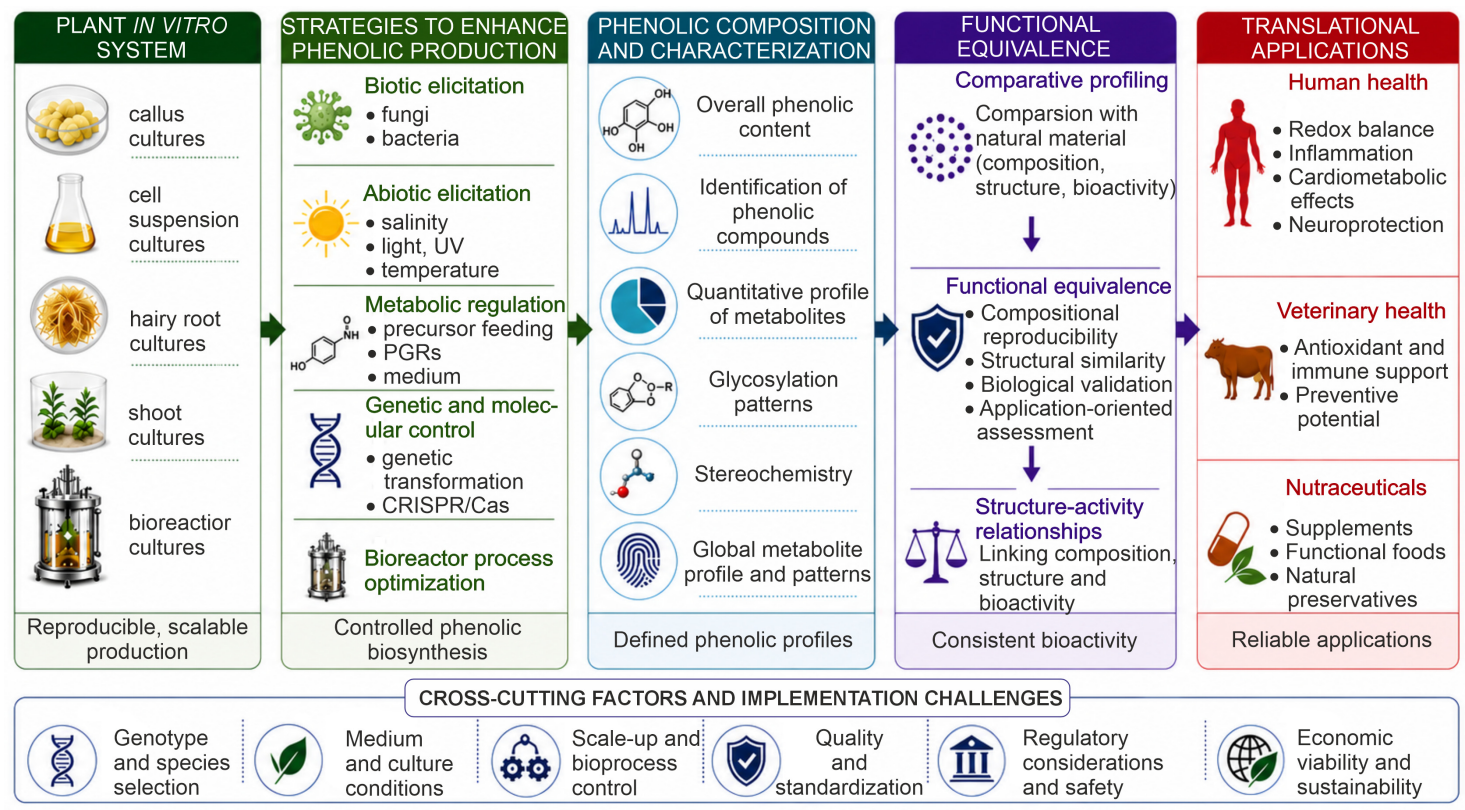
Framework of plant *in vitro* production and functional evaluation of phenolic bioactives. The figure summarizes the main plant *in vitro* culture systems used for phenolic production and the principal strategies applied to enhance biosynthesis, including biotic and abiotic elicitation, metabolic and molecular regulation, and bioreactor process optimization. It also presents the main levels of phenolic characterization and functional comparison required before translational applications can be considered. The final production outcome depends on genotype, culture conditions, process scale, quality control, regulatory requirements, and economic feasibility.

Traditionally, phenolic compounds are obtained from field-grown plants or collected from natural sources; however, environmental variability, developmental stage, and seasonal changes may substantially affect metabolite accumulation and composition, limiting the reproducibility and standardization of plant-derived preparations [5,6,7]. In this context, plant *in vitro* cultures have emerged as controlled production systems that enable the regulation of secondary metabolism and potentially more reproducible production of phenolic compounds under defined cultivation conditions [8] (Figure 1).

Previous reviews have focused mainly on optimization of metabolite production and elicitation strategies [9,10,11]. This review instead examines how culture type and production conditions affect phenolic composition, long-term stability, biological performance, and functional equivalence. It integrates molecular regulation, genetic and genome-editing approaches, bioreactor cultivation, bioavailability, and translational validation.

### Overview of Phenylpropanoid Biosynthesis and Its Regulation in Plant In Vitro Systems

**Figure 2:**
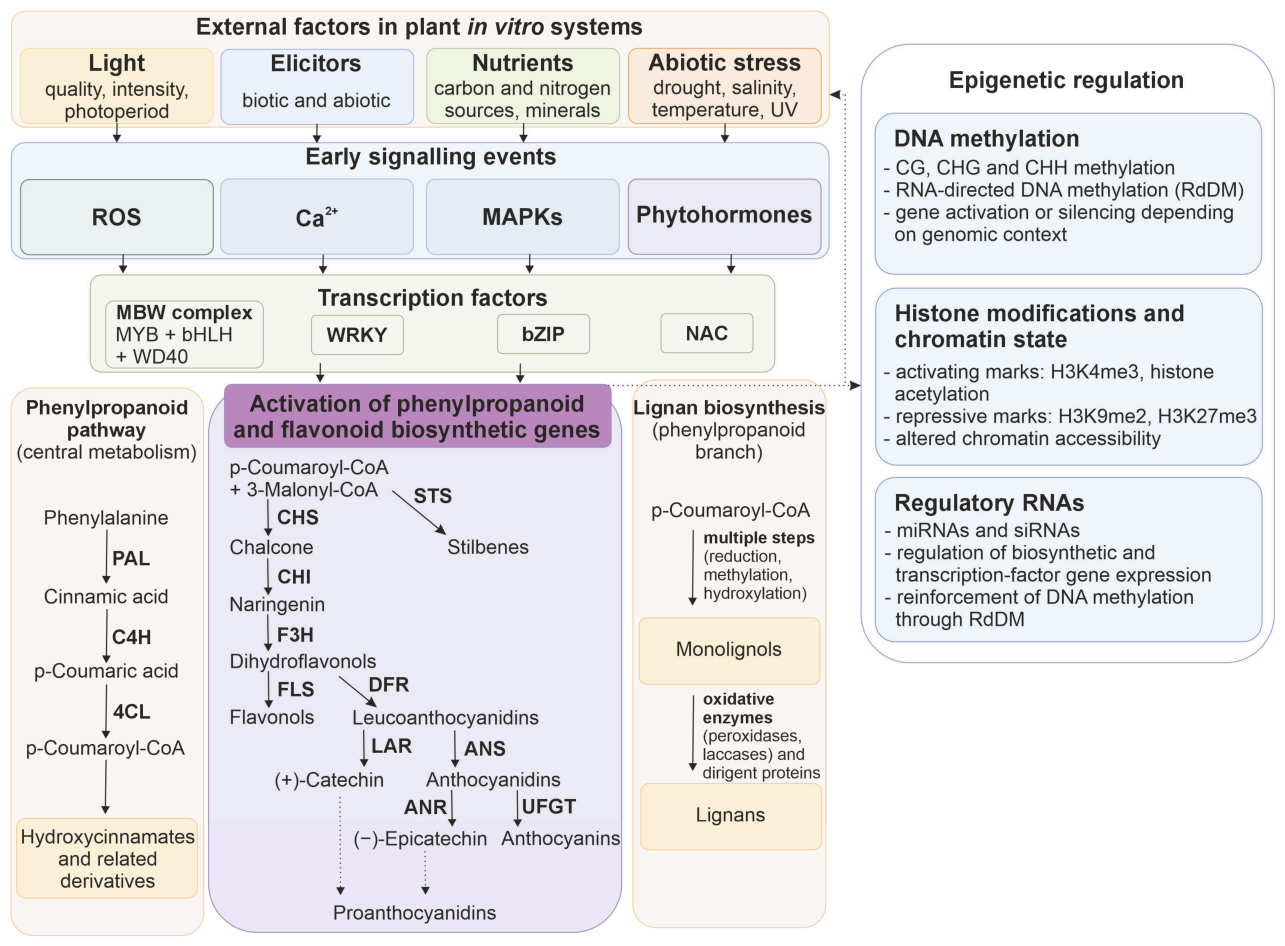
Simplified overview of phenylpropanoid biosynthesis and regulation in plant *in vitro* systems. External factors activate signalling and transcriptional networks controlling the major phenolic branches, while epigenetic mechanisms may affect gene expression and production stability. Solid, dashed, and dotted arrows indicate metabolic conversions, regulatory effects, and incompletely resolved steps in proanthocyanidin formation, respectively.

The phenylpropanoid pathway begins with conversion of phenylalanine into cinnamic acid by phenylalanine ammonia-lyase (PAL; Figure 2). Cinnamic acid is subsequently hydroxylated by cinnamate 4-hydroxylase (C4H) and activated by 4-coumarate-CoA ligase (4CL), leading to formation of p-coumaroyl-CoA, which represents an important intermediate in phenylpropanoid metabolism. Together with other hydroxycinnamoyl intermediates, p-coumaroyl-CoA participates in the formation of several branches leading to phenolic acids, flavonoids, anthocyanins, stilbenes, lignans, and tannins [12,13].

The flavonoid branch is initiated by chalcone synthase (CHS) and chalcone isomerase (CHI), which participate in formation of the basic flavonoid structure. Flavanone 3-hydroxylase (F3H) subsequently produces dihydroflavonols, which serve as precursors of different downstream metabolites [14]. Flavonol synthase (FLS) directs metabolism toward flavonol formation, whereas dihydroflavonol 4-reductase (DFR), anthocyanidin synthase (ANS), and UDP-glucose:flavonoid 3-O-glucosyltransferase (UFGT) participate in anthocyanin biosynthesis and stabilization [15]. Stilbene synthase (STS) competes with CHS for p-coumaroyl-CoA and directs metabolism toward stilbene production [16]. Leucoanthocyanidin reductase (LAR) and anthocyanidin reductase (ANR) participate in proanthocyanidin biosynthesis, whereas dirigent proteins and oxidative enzymes are involved in lignan formation and polymerization [17,18].

Expression of phenylpropanoid and flavonoid biosynthetic genes is regulated mainly by MYB, basic helix-loop-helix (bHLH), and WD40 proteins, which frequently form MYB–bHLH–WD40 (MBW) complexes [19]. WRKY, bZIP, and NAC transcription factors additionally connect secondary metabolism with stress responses, hormonal signalling, and plant development [15,20,21]. In plant *in vitro* systems, light, elicitors, nutrient availability, and abiotic stress may activate signalling pathways involving reactive oxygen species (ROS), calcium ions (Ca^2+^), mitogen-activated protein kinases (MAPKs), and hormones including jasmonate, salicylate, ethylene, and abscisic acid (ABA) [22,23]. These signalling components also regulate other pathways of specialized metabolism, whereas pathway specificity depends mainly on branch-specific enzymes and different combinations of transcription factors. The main regulatory relationships are presented schematically and further discussed in subsequent sections concerning elicitation.

Phenolic biosynthesis is also regulated by DNA methylation, histone modifications, and regulatory small RNAs, particularly during prolonged *in vitro* cultivation, stress adaptation, and cellular dedifferentiation. These changes may affect expression of phenylpropanoid biosynthetic and regulatory genes and consequently influence production stability [13,24]. Tissue differentiation also affects metabolic profiles. Organ and hairy-root cultures may preserve tissue-specific biosynthetic capacities, whereas callus and cell-suspension cultures may show altered or less stable phenolic production [25]. Therefore, developmental and epigenetic status should be considered during optimization of phenolic production and reproducibility in plant *in vitro* systems.

## 2. Plant *In Vitro* Cultures as Bioactive Compound Production Platforms

Plant *in vitro* cultures provide controlled systems for production of phenolic acids, flavonoids, stilbenes, lignans, tannins, and anthocyanins. Compared with field-grown plants, cultivation under defined and aseptic conditions reduces variability associated with climate, seasonality, soil composition, and pathogen pressure. This is particularly relevant for phenolic compounds, whose accumulation and composition depend strongly on environmental and developmental conditions. However, controlled cultivation does not itself ensure high or stable production because the final outcome also depends on culture type, tissue differentiation, medium composition, physiological status, and cultivation duration.

Major plant *in vitro* systems include callus, cell-suspension, hairy-root, adventitious-root, shoot, and other organ cultures. They differ in growth characteristics, metabolic activity, scalability, and degree of tissue differentiation. Differences in phenolic profiles were observed between differentiated and dedifferentiated tissues [26]. Corresponding differences were reported among hairy, adventitious, and seedling roots [27]; selection of the culture system may therefore affect the amount and qualitative composition of accumulated phenolics, as well as their similarity to source plant tissues.

Callus and cell-suspension cultures are used to investigate phenolic regulation and elicitation and to select high-producing cell lines [28]. Callus cultures provide metabolically plastic material for screening and optimization, whereas cell suspensions enable more homogeneous liquid cultivation and are more suitable for process control and scale-up [28,29]. Nevertheless, dedifferentiated cultures may show passage-dependent variability in phenolic accumulation and changes in metabolite profiles during long-term subculture [29,30]. Bioreactor cultivation enables control of aeration, nutrient availability, pH, dissolved oxygen, and mixing. However, successful scale-up requires maintenance of both biomass growth and target-metabolite production [28,31].

Differentiated systems, including hairy-root, adventitious-root, shoot, and microshoot cultures, may retain organ-specific biosynthetic characteristics. Hairy roots combine hormone-independent growth with relatively high genetic stability and may accumulate phenolic compounds at levels comparable to or higher than those found in intact plants [32,33]. Shoot and other organ cultures preserve a higher degree of tissue organization and may retain phenolic profiles qualitatively similar to those found in intact plants [34]. Nevertheless, tissue differentiation does not ensure quantitative or qualitative equivalence, because metabolite levels and relative proportions remain dependent on species, organ type, and culture conditions [33,34].

Table 1 shows that *in vitro* cultures may produce phenolics at levels lower than, comparable to, or higher than those found in intact plant material. High production during one cultivation cycle does not confirm long-term production stability. Long-term phenolic production was demonstrated for selected callus, cell-suspension, and hairy-root lines, whereas in many studies, stability across consecutive passages or production cycles was not evaluated. Their usefulness depends on metabolite composition, scalability, and long-term production stability. Examples of long-term stable and declining phenolic production are discussed in Section 7.1.

Accordingly, differentiated and dedifferentiated cultures should not be considered equivalent production systems [25,26]. Callus cultures are useful for screening, selection of high-producing lines, and testing of elicitation conditions [28]. However, their growth on solid medium limits process monitoring and direct scale-up [28]. Cell-suspension cultures are more suitable for stirred and aerated bioreactors and enable more uniform distribution of nutrients and elicitors [28,29]. However, they may show cell aggregation, sensitivity to shear stress, and passage-dependent changes in metabolite production [28,29,30]. Hairy and adventitious-root cultures may retain root-specific biosynthetic capacity [27,32,33]. Hairy roots, in particular, may show relatively high genetic and metabolic stability [32,33]. However, the branched morphology of root cultures may limit mixing, oxygen transfer, sampling, and biomass recovery at larger scale [32,39]. Shoot and other organ cultures preserve a high degree of tissue differentiation and may retain phenolic profiles qualitatively similar to those of intact plant organs [26,34]. Nevertheless, their organized and heterogeneous structure may limit compatibility with conventional stirred bioreactors and restrict scale-up [28,34]. This distinction is important for phenolic production because dedifferentiated cultures may show greater metabolic plasticity but lower preservation of tissue-specific pathways, whereas differentiated cultures may retain organ-specific biosynthetic capacity more effectively [25,26,32,33,34]. Therefore, no single culture system is optimal for all applications. Selection should depend on the target phenolic profile, degree of tissue differentiation, genetic and metabolic stability, elicitation strategy, bioreactor configuration, downstream recovery, and reproducibility across production batches [25,28,32,33,34].

## 3. Molecular Regulation and Programmable Modulation of Phenolic Biosynthesis in Plant *In Vitro* Systems

Current studies increasingly investigate strategies affecting phenolic biosynthesis in plant *in vitro* cultures, including biotic stimulation, abiotic and metabolic regulation, and molecular approaches modifying metabolite composition.

Under *in vitro* conditions, these regulatory levels act as a connected response. Light, nutrient availability, osmotic or oxidative stress, and biotic or abiotic elicitors may activate ROS, Ca^2+^, MAPK, and hormonal signalling. These signals may affect MYB–bHLH–WD40 complexes and other regulators, including WRKY, bZIP, and NAC, and consequently change the expression of genes encoding PAL, C4H, 4CL, CHS, CHI, FLS, DFR, ANS, and other branch-specific enzymes. As recently summarized by Ren et al. [19], late modifications, transport, and sequestration also affect final flavonoid accumulation, localisation, stability, and biological properties. The final response depends on culture type, developmental status, treatment concentration, timing, and exposure duration. Moderate stimulation may increase phenolic accumulation, whereas excessive or prolonged stress may reduce biomass and total metabolite yield. Molecular responses should therefore be evaluated together with treatment conditions, compound-specific profiles, and biomass productivity [9,10,11,19,20,21,22,23].

### 3.1. Microbial Elicitation and Plant–Microorganism Interactions

Plant–microorganism interactions activate defence-related signalling pathways that regulate phenylpropanoid metabolism and accumulation of phenolic compounds. Plant *in vitro* cultures provide controlled systems for studying these processes and for evaluating microbial elicitors as regulators of phenolic production.

Arbuscular mycorrhizal fungi (AMF), plant growth-promoting rhizobacteria (PGPR), rhizobia, and endophytes are frequently associated with regulation of phenolic metabolism [40,41]. AMF form root symbioses that support nutrient acquisition, PGPR colonize the rhizosphere and promote plant growth, rhizobia establish nitrogen-fixing symbioses with legumes, and endophytes inhabit internal plant tissues without causing disease. Inoculation with AMF, including *Funneliformis mosseae* and *Rhizophagus intraradices*, was associated with increased phenolic accumulation, particularly under drought and salinity stress [42,43]. PGPR belonging to *Rhizobium*, *Azospirillum*, *Bacillus*, and *Pseudomonas* may affect phenolic biosynthesis through modulation of phytohormonal and defence-related signalling [41]. In *Medicago truncatula*, inoculation with *Pseudomonas fluorescens* Ms9N or *Stenotrophomonas maltophilia* Ll4 caused strain-, tissue-, and time-dependent changes in the expression of *Mt*PAL, *Mt*MYB, and *Mt*WRKY genes associated with phenylpropanoid-related defence responses [44]. In legumes, flavonoids also participate in signalling associated with nodulation and plant–rhizobia interactions. However, the response depends on plant species, developmental status, microbial strain, and cultivation conditions. Representative bacterial groups and their reported effects on phenolic metabolism are summarized in Supplementary Table S1.

Microbial signals are recognized by pattern-recognition receptors located in the plasma membrane, including receptor kinases and receptor-like proteins that signal through associated receptor kinases and co-receptors such as SOBIR1 and BAK1 [45,46]. Signal perception induces early responses, including reactive oxygen species production, Ca^2+^ influx, and activation of mitogen-activated protein kinase cascades [47]. These signalling networks interact with salicylic acid, jasmonate, and ethylene pathways and regulate defence-related transcriptional responses [48,49]. Their activation may enhance phenylpropanoid metabolism and promote the accumulation of defence-related phenolic compounds and phytoalexins [47,49].

Activation of phenylpropanoid metabolism also occurs during interactions with pathogenic microorganisms. These responses are associated with accumulation of phenolic compounds involved in antioxidant defence, antimicrobial activity, lignification, and phytoalexin production. Such changes were reported in *Solanum lycopersicum*, *Oryza sativa*, and *Triticum aestivum* [50,51,52].

Increased accumulation of phenolic compounds was reported after treatment with salicylic acid, methyl jasmonate, yeast extract, and pectin in cultures of *Hypericum perforatum*, *Oryza sativa*, and *Salvia przewalskii* [53]. Similar responses, frequently accompanied by increased PAL activity and antioxidant capacity, were observed in cultures of apple, *Washingtonia filifera*, and *Sageretia thea* [54,55,56]. Chitin fragments and other microbial elicitors may also activate defence responses and stimulate accumulation of flavonoids and phenolic acids [57,58]. Gene-expression analyses indicated elicitor-dependent changes in genes associated with defence signalling and phenylpropanoid biosynthesis [59].

Previous exposure to biotic or abiotic stimuli may enable faster or stronger activation of defence pathways during subsequent stress [60,61]. Primed tissues may show earlier activation of phenylpropanoid metabolism and increased activity of PAL, peroxidases, and polyphenol oxidase [61,62,63]. However, the usefulness of priming for metabolite production requires confirmation across consecutive culture cycles.

Responses remain dependent on the plant–microorganism combination, elicitor concentration, treatment duration, culture type, and analytical method. Reproducible application therefore requires defined treatment conditions, analysis of individual phenolic compounds, and confirmation in independent culture batches.

### 3.2. Abiotic and Culture-Related Modulation Strategies

Abiotic factors and culture-related modifications affect phenolic accumulation in plant *in vitro* systems through changes in stress signalling, tissue development, and phenylpropanoid metabolism [51,52]. Salinity, osmotic stress, light quality, UV radiation, temperature, medium pH, and oxidative stress produced measurable changes in phenolic profiles. However, the direction and magnitude of these responses depended on treatment intensity, duration, culture type, and developmental status.

The studies summarized in Table 2 show that phenolic responses differed considerably in duration and magnitude. Some effects were detected within several hours, whereas others developed after several days or weeks. In several cases, increased phenolic accumulation was accompanied by reduced biomass growth or tissue damage. Phenolic concentration should therefore be evaluated together with biomass and total metabolite yield.

Culture-related strategies, including medium pH modification, precursor feeding, and PGR or combined PGR–elicitor treatments, also affected phenolic accumulation and tissue growth [64,65,66]. Their effects were dose-, time-, and culture-dependent, and increased accumulation of selected compounds was not always accompanied by improved biomass production.

Reliable comparison of abiotic and culture-related strategies requires defined treatment intensity and duration, compound-specific analysis, measurement of biomass and total metabolite yield, and evaluation across independent production batches. Short-term induction alone does not demonstrate sustained phenolic production.

**Table 2:** Selected abiotic and culture-related treatments affecting phenolic accumulation in plant *in vitro* systems: treatment conditions, response magnitude, and effects on growth.

Strategy	Plant Species and Culture System	Treatment Conditions and Duration	Quantitative Phenolic Response	Effect on Biomass or Growth	Ref.
**Salinity**	*Glaux maritima* L.; *in vitro* shoot cultures	NaCl: 0, 25, 50, 75, 100, 200, 300, 400, or 500 mM; 40 d.	TPC: 8.9 ± 1.0 mg GAE g^−1^ DW at 75 mM NaCl; TFC: 10.1 ± 1.1 vs. 1.3 ± 0.2 mg RE g^−1^ DW at 100 mM (7.8-fold); HPLC: salinity-dependent increases in protocatechuic acid, astragalin, hyperoside, rutin, and isoquercitrin, with compound-specific maxima at 100–300 mM NaCl	At 300–500 mM NaCl, shoot fresh and dry biomass and shoot/root size decreased; roots were absent at 500 mM	[67]
**Drought/osmotic stress**	*Linum album* Kotschy ex Boiss.; cell-suspension culture	PEG 6000: 0, 2.5, 5.0, 7.5, or 10% (*w*/*v*), added on day 8; 5 d exposure.	Total phenolic acids: 860.48 ± 15.41 vs. 691.44 ± 0.54 µg g^−1^ FW at 2.5% PEG (1.24-fold); gallic acid: 1.69-fold at 10% PEG	Fresh weight: 6.40 ± 0.15 vs. 7.43 ± 0.19 g at 10% PEG; maximum dry weight: 0.69 ± 0.003 vs. 0.41 ± 0.003 g at 5% PEG (1.68-fold)	[68]
**Light quality**	*Verbena officinalis* L.; *in vitro* callus cultures	Red, blue, red/blue LED (70:30), darkness, or fluorescent control; 2, 3, or 4 weeks	Verbascoside: 6716 ± 528 (blue) and 6023 ± 466 mg 100 g^−1^ DW (red/blue); blue, 1.84-fold vs. control. Isoverbascoside: 334 ± 24 and 379 ± 21 mg 100 g^−1^ DW, respectively; red/blue comparable to control (383 ± 26)	Dry-biomass increase at week 4: 17.21-fold (blue), 17.37-fold (red/blue), and 16.55-fold (fluorescent control); maximum: 19.19-fold under blue light at week 3	[69]
**UV radiation**	*Cajanus cajan* (L.) Millsp.; *in vitro* hairy-root cultures	UV-B (3 W m^−2^) applied to 42-day-old hairy roots; sampling at 0, 2, 4, 6, 8, 10, 12, and 24 h	Eight flavonoids: 414.95 ± 50.68 µg g^−1^ DW after 4 h (1.49-fold); cajaninstilbene acid: 6566.01 ± 702.14 µg g^−1^ DW after 10 h (2.31-fold)	Biomass not quantified; UV-B induced oxidative stress and visible hairy-root damage	[70]
**Temperature**	*Lavandula viridis* L’Hér.; *in vitro* cultures	15, 20, 25, or 30 °C; 2 weeks	Rosmarinic acid: 92.6 g kg^−1^ DW at 15 °C (maximum)	Biomass not quantified; 15 and 30 °C were associated with stronger oxidative-stress responses and altered physiological performance	[71]
**Medium pH**	*Physalis peruviana* L.; *in vitro* hairy-root cultures	Initial pH: 4.5, 5.0, 5.5, 6.0, or 6.5; assessment on day 24	At pH 5.5: TPC, 10.31 mg g^−1^ DW; caffeoylputrescine content, 2.25 mg g^−1^ DW; volumetric yield, 28.49 mg L^−1^	Biomass productivity was unchanged at pH 4.5–5.5 but decreased above pH 5.5	[72]
**Oxidative stress**	*Camellia sinensis* (L.) Kuntze; *in vitro* callus cultures	H_2_O_2_: 0.01, 0.1, or 1 mM, applied to 24- or 36-day-old calli grown in light or darkness; assessment after 2 and 48 h	Total phenolics: +55% at 2 h and +63% at 48 h; flavans and soluble proanthocyanidins: approximately twofold at 48 h	No change in biomass increment after 2 or 48 h	[73]
**Precursor feeding**	*Hyoscyamus niger* L.; adventitious *in vitro* root cultures	L-Phenylalanine: 0.25, 0.50, or 1.00 mM; harvest after 1, 3, or 7 d	TPC: 8.82 ± 0.23 vs. 6.85 ± 0.08 mg g^−1^ DW at 0.50 mM L-phenylalanine for 3 days (1.29-fold); gallic acid: 2.67-fold; catechin: up to 4.75-fold	Moderate L-Phe concentrations improved root growth; however, the L-Phe concentration × culture-duration interaction was not statistically significant	[74]
**PGR/elicitor**	*Hyoscyamus niger* L.; adventitious-root and cell-suspension cultures	24-Epibrassinolide (EBL): 0.5, 1, or 2 mg L^−1^, alone or with MJ at 224.3 mg L^−1^ (1 mM), after 7 d pre-culture; roots/cells harvested after 2/3 weeks, respectively	Adventitious roots: TPC 18.01 vs. 5.91 mg g^−1^ with MJ (3.05-fold); cell suspensions: maximum TPC 11.56 mg g^−1^ with 2 mg L^−1^ EBL + MJ	MJ reduced growth, whereas EBL alleviated this effect. At 2 mg L^−1^ EBL, root fresh weight, dry weight, and growth index reached 26.10 g 100 mL^−1^, 1.67 g 100 mL^−1^, and 3.88, respectively	[75]

**Note:** d, days; DW, dry weight; EBL, 24-epibrassinolide; FW, fresh weight; GAE, gallic acid equivalents; h, hours; HPLC, high-performance liquid chromatography; LED, light-emitting diode; L-Phe, L-phenylalanine; MJ, methyl jasmonate; PEG, polyethylene glycol; PGR, plant growth regulator; RE, rutin equivalents; TFC, total flavonoid content; TPC, total phenolic content; UV-B, ultraviolet-B radiation; *w*/*v*, weight per volume.

### 3.3. Genetic and Genome-Editing Approaches for Phenolic Pathway Modulation

Genetic modification of phenolic biosynthesis includes manipulation of structural genes, transcriptional regulators, and reactions competing for the same pathway substrates. Overexpression and RNA interference are mainly used to increase the activity of selected biosynthetic steps or to reduce metabolic competition. CRISPR-based approaches enable targeted disruption of genes or activation of their transcription. Structural genes such as *CHS*, *HQT*, *RAS*, and *CYP98A14* regulate defined reactions or branch points, whereas MYB, bHLH, WD40, and WRKY transcription factors may regulate several pathway genes at the same time. Evidence comes from both production-oriented *in vitro* cultures and regenerated plants; the former directly demonstrate controlled metabolite production, whereas the latter mainly reveal pathway regulation and metabolic-flux redistribution.

Structural-gene modification was directly evaluated in *Salvia miltiorrhiza* hairy-root cultures. Co-expression of *tyrosine aminotransferase* (*TAT*) and *4-hydroxyphenylpyruvate reductase* (*HPPR*) resulted in rosmarinic acid and lithospermic acid B production of 906 and 992 mg/L, respectively, corresponding to 4.3- and 3.2-fold higher than those observed in wild-type hairy roots. Suppression of the *HPPD* gene, which encodes an enzyme competing with HPPR for 4-hydroxyphenylpyruvate, also increased phenolic acid production [76]. In the same culture system, RNAi-mediated silencing of *CHS* reduced flavonoid accumulation and redirected metabolic flux toward phenolic acids, particularly when combined with salicylic acid treatment [65]. In another study, separate overexpression of *rosmarinic acid synthase* (*RAS*) or *CYP98A14* increased phenolic acid content in *S. miltiorrhiza* hairy roots by more than threefold [66]. These results indicate that modification of consecutive pathway steps or a reduction in a competing reaction may be more effective than overexpression of one upstream enzyme. However, metabolite production differed among independently transformed lines, indicating that transgene integration and expression level remain important sources of variability.

Transformation with *Agrobacterium rhizogenes* may induce broader metabolic changes through bacterial T-DNA integration. In *Ocimum basilicum*, transformation with three *A. rhizogenes* strains increased the expression of several phenylpropanoid-related genes, with strain ATCC 39207 producing particularly high transcript levels of *CAD*, *C4H*, *TAT*, *FLS*, *EGS*, *HPPR*, *PAL*, and *RAS*. Rosmarinic acid, eugenol, chicoric acid, and rutin increased, whereas chlorogenic acid, caffeic acid, cinnamic acid, quercetin, vanillic acid, and methyl chavicol did not change significantly [77]. Because the analyses concerned leaves of plants with transformed root systems rather than isolated hairy-root cultures, the study demonstrates strain- and compound-dependent pathway modulation but does not directly evaluate a hairy-root production system.

In potato, RNA interference-mediated suppression of *hydroxycinnamoyl-CoA quinate hydroxycinnamoyl transferase* (*HQT*) reduced chlorogenic acid content by more than 90%. Smaller decreases in total phenolics and antioxidant capacity, together with increased caffeoyl polyamines and changes in anthocyanins and flavonols, indicated redistribution of phenylpropanoid flux. Reduced PAL activity and an early-flowering phenotype further showed that *HQT* suppression affected both metabolism and development [78]. Although the transgenic lines were initially regenerated in tissue culture, the main metabolic and phenotypic analyses were performed on potted plants grown under greenhouse conditions rather than in a production-oriented *in vitro* culture. The study therefore provides evidence of partial and organ-dependent metabolic rerouting following *HQT* suppression.

In tomato, CRISPR/Cas9 knockout of *SlHQT* almost completely eliminated chlorogenic acid and other caffeoylquinic acids, while increasing several hydroxycinnamoyl-glucose derivatives and some flavonoids. Condition-dependent upregulation of *AN1*, *AN2*, *HY5*, and *PIF1a* also indicated additional transcriptional reprogramming [79]. In another study, a CRISPR activation system based on dCas12a fused to the catalytic SET domain of a histone methyltransferase was targeted to the *SlPAL2* promoter. Unlike conventional CRISPR editing, this approach did not cleave DNA but activated SlPAL2 through targeted epigenetic modification. Its expression increased approximately 2–2.5-fold in non-infected plants and up to 5.5-fold after infection with *Clavibacter michiganensis*. Lignin accumulation also increased markedly and reached 42.7% in infected edited plants. This increase was associated with reduced bacterial load and lesion development, without clear negative effects on plant height or fruit weight [80]. Both studies involved *in vitro* transformation and regeneration, but the main analyses were performed on regenerated whole plants rather than in production-oriented cell or organ cultures.

Transient co-expression experiments in strawberry fruits showed that combinations of *FaMYB5* or *FaMYB10* with the bHLH factor *Fa*EGL3 and the WD40 proteins *Fa*LWD1 or *Fa*LWD1-like significantly increased anthocyanin accumulation relative to the empty-vector control [81,82]. In contrast, *Fa*EGL3–*Fa*LWD1/*Fa*LWD1-like combinations lacking the MYB component caused only slight increases in anthocyanin accumulation [81]. *Fa*MYB5-based regulation preferentially promoted proanthocyanidin biosynthesis through activation of the LAR branch, whereas *Fa*MYB10 affected a broader group of genes associated with anthocyanin biosynthesis [81,82]. Although the main co-expression experiments were conducted in fruits rather than in production-oriented *in vitro* cultures, they demonstrated that the composition of the MYB–bHLH–WD40 complex strongly influences both the magnitude and pathway specificity of the metabolic response.

More direct evidence was recently obtained in *S. miltiorrhiza* hairy roots. Overexpression of the WRKY transcription factor *SmWRKY40* increased rosmarinic acid, salvianolic acid B, and lignin accumulation, as well as hairy-root growth. In contrast, CRISPR/Cas9-mediated knockout of *SmWRKY40* reduced phenolic acid accumulation, inhibited root growth, and affected xylem development. Expression analysis showed that *SmWRKY40* regulated genes including *SmRAS*, *SmCYP98A14*, and *SmCCR2*, while protein–DNA interaction assays confirmed its binding to promoters of genes associated with phenylalanine and tyrosine metabolism [83]. This study is particularly relevant to plant *in vitro* production because both overexpression and genome editing were evaluated directly in transformed hairy-root cultures. It also demonstrated that modification of a transcription factor may influence metabolite production together with hairy-root growth and xylem development.

Metabolic compensation, substrate competition, feedback regulation, and effects on tissue growth may change both the amount and composition of phenolic products. Modification of structural genes generally produced more defined metabolic changes, whereas transcription-factor engineering affected several pathway branches and developmental processes at the same time. However, most of the studies discussed above examined selected transformed lines during one experimental period, and stability across repeated subcultures or production cycles was not evaluated. Moreover, the edited cultures were not tested under bioreactor conditions. Therefore, evaluation of molecularly modified production systems should include compound-specific metabolite profiling, biomass productivity, comparison of independent lines, and monitoring of production stability. Integration of transcriptomic and metabolomic data may also help distinguish the direct effect of the modified target from secondary redistribution of metabolic flux.

## 4. Functional Equivalence of *In Vitro*-Derived vs. Natural Phenolics

### 4.1. Structural Determinants of Equivalence

Comparative studies indicate that selected plant *in vitro* systems may retain the capacity for phenolic biosynthesis, although the concentrations, relative proportions, and presence of individual metabolites strongly depend on culture conditions and tissue differentiation. In *Ageratina pichichensis*, wild plants, *in vitro* plants, callus cultures, and cell suspensions contained phenolic compounds but showed different quantitative and qualitative profiles. Callus cultures accumulated higher levels of total phenolics and flavonoids than wild plants, whereas 12-day cell suspensions contained approximately 2.0–2.7 times more total flavonoids than wild plants. *In vitro* cultures also produced epicatechin, caffeic acid, and p-coumaric acid, which were not detected in wild material, although their antioxidant activity remained lower [84]. In *Bacopa monnieri* shoot cultures, chlorogenic, neochlorogenic, and caffeic acids remained detectable under different *in vitro* conditions, although their concentrations varied substantially [85]. This example indicates preservation of selected metabolites within the culture system rather than direct equivalence with naturally grown plants. Preservation of phenolic biosynthetic capacity therefore does not exclude substantial compositional changes associated with tissue organization and differentiation. Structural equivalence does not require identical metabolite composition but requires preservation of chemical features relevant to the intended biological function. Comparable biological performance should be evaluated separately using appropriate functional assays.

In *Viola odorata*, biomass cultivated in a stirred-tank bioreactor contained higher levels of total phenolics and flavonoids than natural material, reaching 8.42 versus 3.8 mg GAE/g DW and 5.76 versus 3.1 mg QE/g DW, respectively [86]. However, its antioxidant activity was lower, and equivalence of individual phenolic compounds was not demonstrated. These results support preservation of phenolic biosynthetic capacity rather than full chemical or functional equivalence.

Similarity between phenolic compounds produced under *in vitro* conditions and those occurring naturally depends not only on preservation of the core metabolite structure but also on post-biosynthetic modifications. Glycosylation may influence the physicochemical properties and biological activity of phenolic compounds, including their solubility, stability, and interactions with biological targets, thereby affecting their final biological performance [87,88]. Plant *in vitro* cultures may be used to investigate post-biosynthetic modifications through changes in culture conditions and elicitation strategies. Enhanced accumulation of phenolic compounds accompanied by changes in glycosylation patterns was reported in shoot cultures of *Lychnis flos-cuculi* [89], indicating that culture conditions may influence not only total phenolic levels but also the chemical forms in which these metabolites accumulate. Depending on the phenolic class, acylation may affect anthocyanin stability, whereas the extractability and biological activity of proanthocyanidins may depend on their structural characteristics, including the degree of polymerization [90,91,92]. Similarly, changes in environmental conditions modified individual metabolite profiles in *Hypericum perforatum* hairy-root cultures, indicating regulation of specific phenolic compounds rather than changes limited to total phenolic content [93].

Stereochemical variation generated during biosynthesis may also affect the final biological properties of phenolic compounds. Because phenolic metabolism is enzyme-dependent, stereochemical configuration may influence antioxidant activity and interactions with biological targets [94,95]. Detection of the same compound class or core molecular structure may therefore be insufficient to demonstrate equivalent biological performance.

Overall, phenolic compounds obtained under *in vitro* conditions may retain major structural features of their naturally occurring counterparts. Nevertheless, differences in abundance, relative composition, glycosylation, and stereochemical configuration may affect biological activity and should therefore be considered during evaluation of functional equivalence.

### 4.2. Functional Performance of In Vitro-Derived Phenolics

Functional equivalence requires comparable biological performance of *in vitro*-derived and reference plant material under standardized experimental conditions. Chemical antioxidant assays measure physicochemical properties, such as radical-scavenging and reducing capacity, rather than biological activity in cells or organisms. Therefore, compared samples should be prepared using the same extraction procedure and tested within the same concentration range, with the same controls, reaction conditions, and calculation methods. In the ABTS assay, the measured response depends on antioxidant concentration and reaction time [96], while the extraction solvent may affect total phenolic and flavonoid contents, as well as the results of DPPH, ABTS, reducing-power, FRAP, and H_2_O_2_-scavenging assays [97,98]. Apparent differences between samples may therefore result from extraction and assay conditions rather than from differences in their antioxidant capacity.

Biological performance cannot be predicted only from phenolic content because individual compounds may show different cellular effects. Cell-based assays showed compound-specific responses: acteoside and diplacone were active, whereas morusin was inactive, and pomiferin combined mild activity with cytotoxicity [99]. Although this study did not directly compare *in vitro*-derived and natural material, it illustrates why phenolic content alone cannot predict cellular performance.

Comparative studies show two different outcomes. In some cases, higher phenolic accumulation or antioxidant capacity is accompanied by substantial differences in chemical composition or biological performance compared with the natural plant material. In other cases, acceptable functional comparability may be demonstrated for a defined biological endpoint despite differences in metabolite composition.

The first outcome was reported for several plant *in vitro* systems. In *Thalictrum foliolosum*, *in vitro*-propagated roots contained higher total phenolic and flavonoid levels than wild-collected roots. However, the relative amounts of individual metabolites differed between the two materials. Chlorogenic, gallic, vanillic, and caffeic acids were more abundant in *in vitro*-propagated roots, whereas catechol, anisic acid, and rutin were more abundant in wild roots. The chloroform fraction of *in vitro*-propagated roots also showed stronger DPPH-scavenging activity than the corresponding wild-root fraction, with IC_50_ values of 23.36 and 50.62 µg mL^−1^, respectively [100]. These results indicate increased phenolic production and stronger activity in the applied chemical assay, but they do not confirm preservation of the complete chemical profile or broader functional equivalence.

A different relationship was observed in *Ageratina pichichensis*. Callus cultures accumulated higher total phenolic and flavonoid levels than wild plants, whereas 12-day cell-suspension cultures contained approximately 2.0–2.7-fold more total flavonoids. Epicatechin, caffeic acid, and p-coumaric acid were detected in the *in vitro* cultures but not in the wild material. Nevertheless, callus and cell-suspension extracts showed lower antioxidant activity than the wild plant in the DPPH, ABTS, and TBARS assays [84]. This example indicates that increased total phenolic or flavonoid content may be accompanied by changes in chemical composition and lower antioxidant activity.

A similar result was reported for *Viola odorata*. Biomass cultivated in a stirred-tank bioreactor contained higher total phenolic and flavonoid levels than natural petiole material, reaching 8.42 versus 3.8 mg GAE g^−1^ DW and 5.76 versus 3.1 mg QE g^−1^ DW, respectively. However, only 18 of approximately 60–70 compounds detected in the bioreactor-derived extract were also detected in the natural plant extract. The natural plant extract also showed stronger DPPH-scavenging activity, with an IC_50_ value of 647 µg mL^−1^, compared with 2901 µg mL^−1^ for the stirred-tank-derived biomass extract [86]. These results show that higher total phenolic and flavonoid contents do not necessarily indicate similarity in the complete metabolite profile or antioxidant performance.

In contrast, acceptable functional comparability for a defined biological endpoint was demonstrated in *Clinopodium vulgare*. Aqueous extracts from corresponding organs of *in vitro*-cultivated and wild-growing plants were prepared using the same procedure. The extracts were tested using the MTT assay against HeLa, HT-29, and MCF-7 tumor cells after 24 and 48 h, with non-tumor HaCaT keratinocytes included to evaluate selectivity. Despite qualitative and quantitative differences in their phenolic profiles, extracts from corresponding organs showed comparable concentration- and time-dependent effects on tumor-cell viability [101]. This study therefore supports functional comparability for the defined MTT endpoint, but it does not confirm general equivalence for other biological activities or applications.

These examples indicate that functional differences may result from variation in the identity, relative abundance, and chemical forms of individual phenolics. Culture conditions and duration may modify metabolite profiles [102], while the glycosylation, acylation, polymerization, and stereochemical differences discussed in Section 4.1 may additionally affect compound stability, solubility, and interactions with biological targets. Activity in complex mixtures may also depend on additive, synergistic, or antagonistic interactions among phenolics [103]. The surrounding matrix and interactions with carbohydrates, lipids, and proteins may further modify compound release, bioaccessibility, stability, and activity [104,105]. Thus, preparations with similar total phenolic contents may differ in biological performance because of differences in metabolite proportions and matrix composition.

Interpretation of biological activity also depends on the experimental model. Antioxidant effects should, where relevant, be confirmed in cellular oxidative-stress models by measuring intracellular reactive oxygen species, oxidative damage, antioxidant-enzyme activity, or changes in cellular antioxidant-response pathways, together with cell viability [106]. Anti-inflammatory activity requires evaluation of inflammatory mediators, related enzymes, or signalling pathways [107], whereas antibacterial activity should be tested using standardized susceptibility methods and quantitative endpoints such as the minimum inhibitory concentration [108]. Conclusions concerning biological activity should therefore remain limited to the specific endpoint and experimental model used [106,108,109].

Meaningful comparison also requires clear information on culture type, degree of tissue differentiation, culture conditions, elicitor treatment, and culture or passage duration. Metabolite production may depend on specific cell types, organelles, developmental stages, medium composition, and elicitation conditions [9]. Prolonged culture maintenance and repeated subculturing may also be associated with epigenetic changes and a progressive decline in secondary-metabolite production [110]. Functional comparability should also be reproducible across independently produced culture batches and supported by consistent chemical fingerprints, marker-compound levels, and biological assay results.

Functional equivalence should be established for a defined biological function rather than treated as a general property of an extract. It requires compound-specific profiling, batch-to-batch reproducibility, and directly comparable biological testing [111,112,113,114,115,116]. Higher total phenolic content or stronger activity in a single chemical assay is not sufficient. Broader health-related or therapeutic claims additionally require evaluation of bioavailability, metabolism, toxicity, and in vivo efficacy, as discussed in Section 5 and Section 6.

## 5. Translational Evidence and Biological Validation of Phenolic Compounds

The translational relevance of phenolic compounds depends not only on biological activity but also on metabolite composition, batch-to-batch reproducibility, extraction and formulation procedures, stability, and biological exposure [117,118,119]. Plant *in vitro* systems may reduce environmental variability and enable control of medium composition, growth regulators, culture duration, and harvest time [102]. However, controlled cultivation does not itself ensure a chemically uniform or biologically effective preparation. Standardization must therefore be established for a defined species, genotype, culture system, and production protocol.

Direct human or veterinary evidence concerning standardized phenolic preparations produced specifically in plant *in vitro* systems remains scarce. The most direct evidence currently available for *in vitro*-derived preparations comes from chemical assays, microbial tests, and cellular models. As shown for *Ageratina pichichensis* in Section 4.2, increased phenolic accumulation did not result in proportionally greater antioxidant activity [84]. In a manipulated cell culture of *Mirabilis jalapa*, bioassay-guided fractionation identified phenolic compounds active against *Candida albicans*, with IC_50_ values of 25 and 48 µg mL^−1^ for the two principal antifungal constituents [120]. These results confirm that plant cell cultures can produce biologically active compounds, but chemical or microbial assays alone do not demonstrate therapeutic efficacy, systemic exposure, or safety in vivo.

More direct mechanistic evidence is available from cell-based studies. Fractions obtained from hairy roots of *Dracocephalum moldavica* contained caffeic acid, rosmarinic acid, and salvianolic acid B. At 10 mg mL^−1^, the caffeic acid-rich fraction reduced LPS-induced NF-κB reporter activation by 53% and IL-1β production by 47% in differentiated THP-1 monocyte-based assays, without suppressing the growth of L929 fibroblasts [121]. An extract from hairy roots of *Portulaca oleracea*, tested at 0.1 mg mL^−1^ in LPS-stimulated rat microglial cells, reduced nitric oxide production and expression of *iNOS* and *TNF-α*, without a prominent reduction in cell viability [122]. These studies directly connect a defined *in vitro* production system with chemical characterization and measurable cellular effects. However, interpretation remains limited by the use of concentrated extracts, short exposure periods, individual cellular models, and the absence of pharmacokinetic and in vivo dose–response data.

Evidence relevant to human applications is mainly indirect. Reviews describe effects of phenolic compounds on inflammatory and antioxidant pathways, including NF-κB, Nrf2, cyclooxygenase, and nitric oxide-related responses, but these analyses combine isolated compounds, conventional extracts, cell models, and animal studies [123]. Evidence concerning chlorogenic acids includes epidemiological associations between coffee consumption and reduced incidence of type 2 diabetes, whereas acute intervention studies show limited or inconsistent effects on glucose and insulin responses [124]. A chemically characterized extract from wild *Hypericum perforatum* showed antioxidant, antimicrobial, and cytotoxic activity, but activity was also observed in non-cancerous epithelial cells, and clear tumor selectivity was not demonstrated [125]. These studies support the biological potential of phenolic compounds but do not directly validate preparations produced in plant *in vitro* systems or establish clinical efficacy.

Veterinary evidence is more extensive at the animal level, although it also concerns conventional plant material, tannin-containing feeds, or isolated compounds. In sheep, dietary tannin-rich preparations reduced gastrointestinal parasite egg counts and affected physiological responses during *Haemonchus contortus* infection [126,127]. Other studies showed that tannic acid and grape-seed procyanidins modified lipid parameters, short-chain fatty acids, and intestinal microbial communities, although these effects should not be interpreted as a general improvement in gastrointestinal health [128]. In broilers, a chlorogenic acid complex improved antioxidant responses and reduced intestinal damage in a chemically induced stress model [129]. Chlorogenic acid also reduced mortality, caecal *Escherichia coli* counts, inflammatory mediators, and intestinal oxidative damage after a challenge with avian pathogenic *E. coli* [130]. These findings provide animal-level evidence for selected phenolic preparations, but the tested materials were not produced in plant *in vitro* systems and therefore do not directly confirm the translational value of *in vitro*-derived phenolics.

Translational validation requires defined analytical and biological criteria. Individual compounds should be identified and quantified using validated chromatographic methods, and acceptable concentration ranges should be established across independent production batches. Extraction yield, purity, residual solvents, formulation stability, and degradation during storage should also be evaluated. Functional comparisons should include *in vitro*-derived and conventionally grown material from the same species and, where possible, the same genotype, processed using equivalent extraction procedures and tested at composition-matched doses. Oral preparations additionally require bioavailability assessment, as discussed in Section 6 [117,118,119]. Human development requires toxicity, pharmacokinetic, dose-ranging, and controlled clinical studies, whereas veterinary implementation requires species-specific safety studies and validation under relevant production conditions.

At present, chemical and cell-based studies demonstrate that plant *in vitro* cultures can produce phenolic compounds and extracts with antioxidant, antifungal, or anti-inflammatory activity [84,120,121,122]. Animal studies provide indirect support for selected tannin and chlorogenic acid preparations but do not validate *in vitro*-derived products [127,130]. Direct clinical evidence for standardized *in vitro*-derived phenolic preparations remains lacking. Real-world implementation has occurred mainly in the cosmetic and, less frequently, food sectors. However, reported commercial products generally contain complex plant cell-culture extracts rather than standardized phenolic preparations with demonstrated functional equivalence or clinical efficacy [131]. Overall, current evidence supports improved production control rather than demonstrated functional or clinical superiority over conventionally sourced plant material [102].

## 6. Bioavailability as a Translational Bottleneck

Bioaccessibility and bioavailability are major determinants of the translational performance of orally administered phenolic preparations. Bioaccessibility refers to the fraction released during gastrointestinal digestion and potentially available for intestinal absorption, whereas bioavailability concerns the extent to which an ingested compound or its metabolites becomes available in the systemic circulation [118,132]. Limited aqueous solubility, chemical instability, and transformation during digestion may reduce oral availability of phenolic compounds [118,133]. Following ingestion, these compounds may undergo glucuronidation, sulfation, methylation, and microbiota-dependent biotransformation, with the resulting metabolites entering systemic circulation or being excreted [118].

Relative metabolite composition and structural features, including glycosylation and, particularly for anthocyanins, acylation, may influence phenolic stability, intestinal absorption, and subsequent metabolism [134,135]. The composition and physical organization of the surrounding matrix may additionally affect interactions with other constituents and the release and bioaccessibility of phytochemicals during digestion [136]. Consequently, comparable phenolic classes or total content do not ensure comparable gastrointestinal release, absorption, or metabolism.

Simulated gastrointestinal digestion provides information on compound transformation, release, solubilization, and bioaccessibility but does not establish intestinal absorption or systemic exposure [132]. When combined with differentiated intestinal epithelial models, such as Caco-2 monolayers, it may provide estimates of cellular uptake and transepithelial transport, although whole-body disposition is not reproduced [137]. Claims concerning systemic exposure require in vivo pharmacokinetic evidence, whereas conclusions concerning human bioavailability require pharmacokinetic or metabolite data obtained in human studies, as demonstrated in studies of flavan-3-ols and their circulating and urinary metabolites [132,138].

Biopolymer- and lipid-based carriers, including emulsion-based systems, have been investigated to protect plant-derived bioactive compounds and modify their release [139]. Alginate encapsulation may reduce gastric release and promote intestinal bioaccessibility [140], whereas solid lipid nanoparticles protected rosmarinic acid during simulated gastrointestinal digestion and provided controlled release [141]. However, these outcomes demonstrate improved formulation performance rather than systemic bioavailability or in vivo efficacy. Evaluation of *in vitro*-derived phenolic preparations should therefore combine chemical profiling with appropriate digestion and intestinal-transport models.

## 7. Challenges and Limitations

### 7.1. Biological and Technological Limitations

Despite considerable development of plant *in vitro* technologies, reproducible phenolic production remains challenging. Phenolic accumulation depends on culture conditions, nutrient availability, elicitor treatment, developmental status, and culture age, which may cause passage-to-passage and batch-to-batch variation in biomass growth and metabolite production [142]. Long-term cultivation may also induce somaclonal, genetic, or epigenetic changes affecting metabolic activity. Sweet orange cultures maintained *in vitro* for 30 years showed increased CHH-type DNA methylation and variation associated with dedifferentiation, although phenolic production was not directly evaluated [143].

Direct instability was demonstrated in the LSC-5Y cell-suspension line of *Camellia sinensis*, in which total catechin production decreased by approximately 61% during 2.5 years [144]. In contrast, rosmarinic acid production in *Salvia officinalis* hairy roots remained stable for at least 23 passages, corresponding to almost two years [145]. Long-term production was also reported for caffeoylquinic acids in *Scorzonera radiata* callus for more than 20 years, anthocyanins in engineered *Nicotiana tabacum* cell suspensions for more than 10 years, and phenolic acids in *Dracocephalum forrestii* hairy roots for at least 3.5 years [35,37,38]. These examples show that stability is possible but must be confirmed separately for each culture line and target metabolite.

Production stability should include biomass productivity, concentration and volumetric yield of target compounds, and preservation of the broader metabolite profile. Stable biomass growth or total phenolic content alone may conceal changes in individual compounds. Stability should therefore be evaluated across consecutive passages, independent batches, or production cycles using marker-compound quantification and chemical fingerprinting.

For elicited or molecularly modified cultures, reproducibility should also be confirmed after repeated treatment and prolonged subculture. Short-term stimulation does not demonstrate sustained production, whereas engineered cultures require confirmation that both the introduced trait and metabolite phenotype remain stable.

Scale-up is not limited to increasing culture volume because changes in mixing and mass transfer may affect both biomass growth and phenolic production [28,146]. Higher biomass density, increased medium viscosity, and cell aggregation may reduce mixing and oxygen transfer, leading to spatial differences in oxygen and nutrient availability and less uniform distribution of elicitors within the bioreactor [28,86,146]. Increasing agitation or aeration may improve mass transfer, but it may also increase shear stress and damage sensitive plant cells. Therefore, the balance between oxygen availability and hydrodynamic stress depends on culture morphology, biomass density, and bioreactor configuration [28,86,146].

Elicitation may introduce additional variability during scale-up. Its effects depend on elicitor concentration and treatment duration, while the time of elicitor addition should be coordinated with biomass accumulation [75,147]. At larger scale, uniform exposure of the biomass to the elicitor also depends on mixing and mass transfer [28,146]. Conditions optimized in shake flasks may therefore not produce the same response in larger bioreactors. Non-uniform exposure may contribute to variation in target-metabolite accumulation and phenolic composition. Elicitation conditions should therefore be re-optimized after scale-up and confirmed across independent production batches.

These upstream limitations are also connected with downstream recovery. Biomass aggregation and structural heterogeneity may complicate representative sampling, harvesting, and biomass disruption, whereas extraction and purification conditions determine the recovery and purity of the target compounds [28,86,148]. Extraction, concentration, and solvent-removal conditions may additionally cause degradation, transformation, or changes in the final phenolic profile [115,149]. Therefore, successful scale-up should be evaluated using biomass productivity, compound-specific and volumetric yields, extraction and recovery efficiency, chemical fingerprints, and reproducibility across independent production batches rather than biomass production alone [113,114,115,146,148,149].

### 7.2. Standardization, Stability, and Economic Feasibility

Application of plant *in vitro* systems requires consideration of upstream cultivation, downstream recovery, product quality, and economic feasibility as connected parts of the same production process. A culture may provide high biomass or phenolic concentration but remain unsuitable for practical application if biomass separation is difficult, extraction efficiency is low, process duration is long, or extensive purification is required [146,148].

Phenolic compounds are sensitive to oxidative and environmental conditions. Therefore, harvesting, biomass disruption, extraction, concentration, solvent removal, and storage may affect the final metabolite composition and biological performance [115,149]. Standardization should include defined cultivation and harvest conditions, extraction procedures, recovery yields, marker-compound quantification, and broader chemical fingerprinting. Where biological activity is relevant to the intended application, appropriate functional assays should also be included. These parameters should be evaluated across independent production batches to determine whether scale-up preserves product composition and performance.

Economic feasibility depends on capital expenditure, operational costs, process duration, energy and solvent consumption, labor requirements, extraction efficiency, product recovery, and quality-control requirements [146,148]. Increased metabolite concentration may not reduce the final production cost if it is accompanied by lower biomass productivity, difficult harvesting, inefficient recovery, or extensive purification. Cost-effectiveness should therefore be evaluated together with volumetric productivity, recovery yield, product quality, and batch-to-batch reproducibility rather than only biomass yield or total phenolic content.

## 8. Future Perspectives and Conclusions

Plant *in vitro* systems can provide stable and scalable platforms for phenolic production, but this has been demonstrated only for selected culture lines and metabolites [31,35,37,38].

Increased phenolic accumulation does not ensure preservation of metabolite composition, biological activity, or functional equivalence. Functional equivalence should be demonstrated for a defined use through reproducible chemical profiles and comparable biological performance rather than assumed from total phenolic content or individual antioxidant assays. The main current advantage of plant *in vitro* technology is improved control of production conditions, whereas direct translational evidence remains limited.

Future studies should evaluate compound-specific and volumetric productivity, stability across passages and independent batches, metabolomic profiles, and application-relevant biological activity. Where translational claims are made, bioavailability, metabolism, safety, and formulation should also be considered. Further progress will depend not only on increasing phenolic yield but also on achieving stable production, reproducible composition, and demonstrated functional equivalence and translational suitability.

## Figures and Tables

**Table 1:** Selected representative plant *in vitro* systems for phenolic production: cultivation scale, quantitative production, comparison with intact plant material, and reported production stability. Values and units are reported as presented in the original studies. Colorimetric estimates of total phenolic and flavonoid contents are not directly comparable with compound-specific chromatographic measurements. Long-term stability refers to phenolic production monitored during prolonged cultivation or across consecutive subcultures.

Culture or Production System	Plant Species	Target Phenolics	Cultivation Scale and Production	Comparison with Intact Plant Material	Culture Duration and Production Stability	Ref.
**Callus cultures**	*Scorzonera radiata* Fisch.	Caffeoylquinic acids, mainly 5-CQA and 3,5-diCQA	100 mL flasks, solid W(B/A) medium; total CQAs: 27.95 ± 1.86 mg g^−1^ DW; 5-CQA: 7.54 ± 0.80 mg g^−1^ DW; 3,5-diCQA: 18.52 ± 1.98 mg g^−1^ DW	Total CQAs 1.35-fold higher than in leaves (27.95 vs. 20.71 mg g^−1^ DW); identical CQA spectrum, but callus lacked leaf flavonoids and dihydrostilbenes	30-day subculture cycle; stable CQA production for >20 years; no declining trend during quantitative monitoring in 2014–2022	[35]
**Cell suspensions**	*Cajanus cajan* (L.) Millsp.	Cajaninstilbene acid and 10 flavonoids	Shake-flask MS culture; total phenolics: 3462.99 µg g^−1^ DW; CSA: 2858.76 µg g^−1^ DW; biomass: 10.03 g L^−1^	Total phenolics slightly higher than in field-grown leaves (3462.99 vs. 3288.81 µg g^−1^ DW); CSA 1.35-fold higher	49-day cycle; long-term stability not assessed	[36]
	*Nicotiana tabacum* cv. Samsun, engineered AmDel/AmRos1 line	Cyanidin 3-*O*-rutinoside and engineered anthocyanins	100 mL shake-flask cultures; AmDel/AmRos1: up to 30 mg C3R g^−1^ DW; AmDel/AmRos1/PhF3′5′H: D3R ≥ 30% of total anthocyanins; AmDel/AmRos1/Sl3AT: ~50% aromatically acylated anthocyanins, mainly C3couR and C3ferR	No direct quantitative comparison; wild-type plants accumulated anthocyanins mainly in petals	Medium renewed every 7 days; production maintained for >10 years without substantial decline	[37]
**Bioreactors**	*Vitis labrusca* L. cv. Concord	trans-Resveratrol, pallidol, δ- and ε-viniferin	Stirred-tank bioreactor, 20 L working volume; resveratrol: 4.23 g L^−1^; δ-viniferin: 0.76 g L^−1^	Not directly compared	18-day production process; scale-up without loss of growth or elicitation efficiency; long-term stability not assessed	[31]
	*Reynoutria japonica* Houtt.	Phenolic acids, catechins and flavonoids	Plantform™ temporary-immersion bioreactor, 500 mL medium; total phenolics: shoots 2638.0, roots 1569.3 mg 100 g^−1^ DW	Higher than soil-grown plants: shoots 2638.0 vs. 2149.3; roots 1569.3 vs. 785.7 mg 100 g^−1^ DW; similar qualitative phenolic profile	5-week cycle; long-term stability not assessed	[34]
**Hairy-root cultures**	*Dracocephalum forrestii*	Rosmarinic acid, salvianolic acid B, caffeic acid	Liquid WPM, laboratory scale; RA: 19.97 mg g^−1^ DW; SAB: 11.7 mg g^−1^ DW	RA 4.25-fold higher than in field-grown roots; SAB lower (11.7 vs. 16.4 mg g^−1^ DW)	30-day cycle; stable production for ≥3.5 years	[38]
	*Agastache rugosa*	RA and other phenolic derivatives	Flasks and bioreactors (NSB, RITA^®^, Plantform^®^); maximum RA in NSB: 9.16 mg g^−1^ DW, approximately 80 mg per cycle	No direct comparison; RA comparable with literature-reported levels in field-grown leaves	17-day cycle; four successive subcultures; long-term stability not assessed	[39]
**Organ cultures—adventitious roots**	*Salvia apiana* Jeps.	Rosmarinic acid	Shake-flask SH culture with IBA; RA: 44.49 mg g^−1^ DW; biomass: 15.74 g DW L^−1^	RA not detected in field-grown roots; *in vitro* roots lacked root-specific diterpenoids	Maximum RA after 33 days; necrosis after ~40 days; long-term stability not assessed	[33]
**Organ cultures—microshoots**	*Schisandra henryi* C.B.Clarke	Phenolic acids and flavonoids	Agar MS culture; total phenolic acids: 840.89 mg 100 g^−1^ DW; total flavonoids: 421.98 mg 100 g^−1^ DW	Phenolic acids 7–9-fold and flavonoids 1.4–2.2-fold higher than in parent-plant leaves	10–30-day cycles; subcultured every 30 days; long-term stability not assessed	[26]

**Note**: 3,5-diCQA, 3,5-dicaffeoylquinic acid; 5-CQA, 5-*O*-caffeoylquinic acid; AmDel, *Antirrhinum majus* DELILA; AmRos1, *Antirrhinum majus* ROSEA1; C3couR, cyanidin 3-*O*-(coumaroyl)rutinoside; C3ferR, cyanidin 3-*O*-(feruloyl)rutinoside; C3R, cyanidin 3-*O*-rutinoside; CQA, caffeoylquinic acid; CSA, cajaninstilbene acid; D3R, delphinidin 3-*O*-rutinoside; DW, dry weight; IBA, indole-3-butyric acid; MS, Murashige and Skoog medium; NSB, nutrient sprinkle bioreactor; PhF3′5′H, *Petunia* × *hybrida* flavonoid 3′,5′-hydroxylase; RA, rosmarinic acid; RITA^®^, temporary immersion bioreactor system; SAB, salvianolic acid B; SH, Schenk and Hildebrandt medium; Sl3AT, *Solanum lycopersicum* anthocyanin 3-*O*-rutinoside-4′′′-hydroxycinnamoyl transferase; WPM, Woody Plant Medium;

## Data Availability

No new data were created or analyzed in this study.

## Supplementary Materials

The following supporting information can be downloaded at: https://www.mdpi.com/article/10.3390/ijms27156996/s1. References [150,151,152,153,154] are cited in the supplementary materials.

## Abbreviations

The following abbreviations are used in this manuscript:

ABTS	2,2′-azino-bis(3-ethylbenzothiazoline-6-sulfonic acid)
AMF	arbuscular mycorrhizal fungi
bHLH	basic helix-loop-helix
CHS	chalcone synthase
CRISPR	clustered regularly interspaced short palindromic repeats
DPPH	2,2-diphenyl-1-picrylhydrazyl
HQT	hydroxycinnamoyl-CoA quinate hydroxycinnamoyl transferase
MJ	methyl jasmonate
MYB	MYB transcription factor
PAL	phenylalanine ammonia-lyase
PEG	polyethylene glycol
PGPR	plant growth-promoting rhizobacteria
PGR	plant growth regulator
RA	rosmarinic acid
RAS	rosmarinic acid synthase
TPC	total phenolic content
WD40	WD40-repeat protein
WRKY	WRKY transcription factor
