# Supplementary material for: Plant In Vitro Production of Phenolic Bioactives: Molecular Regulation, Functional Equivalence and Translational Challenges
Source: Int J Mol Sci. 2026 Aug 4;27(15):6996. doi: 10.3390/ijms27156996 (PMC13466245; doi:10.3390/ijms27156996)
Supplement: Supplementary file 1 [file ijms-27-06996-s001.zip › ijms-4424409-supplementary.pdf]

Table S1. Representative bacterial interactions affecting phenolic biosynthesis and phenylpropanoid-related responses in plants

Bacterial group	Species or strain	Plant species or experimental model	Specific phenolic or phenylpropanoid response	Ref.
**Plant growth-promoting rhizobacteria (*Bacillus*)**	*Bacillus tequilensis* PKDN31 + *Bacillus licheniformis* PKDL10	*Solanum lycopersicum* plants challenged with *Fusarium oxysporum* f. sp. *lycopersici*	Combined seed inoculation increased phenylalanine ammonia-lyase (PAL), polyphenol oxidase, peroxidase, and total phenol accumulation and was associated with induced systemic resistance to *Fusarium wilt*.	[150]
**Plant growth-promoting rhizobacteria (*Pseudomonas*)**	*Pseudomonas fluorescens* VSMKU3054	*Solanum lycopersicum* seedlings challenged with *Ralstonia solanacearum*	Increased PAL, peroxidase (POX), polyphenol oxidase (PPO), lipoxygenase (LOX), and total phenol content. The highest total phenol content and POX activity occurred 24 h after pathogen challenge; PAL and PPO peaked at 36 h and LOX at 48 h.	[151]
**Symbiotic rhizobium**	*Sinorhizobium meliloti* KK13	*Medicago truncatula* seedlings; roots and leaves sampled 24, 72, and 168 h after inoculation	Modulated *MtPAL2*–*MtPAL4* expression, PAL activity, and total phenolic content. *MtPAL4* transcript abundance in leaves increased approximately 4-fold at 24 h; responses differed between tissues and sampling times.	[152]
**Other plant growth-promoting bacterium**	*Paenibacillus borealis* KK4	*Medicago truncatula* seedlings; roots and leaves sampled 24, 72, and 168 h after inoculation	Had relatively little effect on MtPAL2–MtPAL4 expression but altered PAL activity and total phenolic content in a tissue- and time-dependent manner. Root PAL activity increased by 88% at 168 h. Total phenolic content increased by 267% and 49% in roots at 72 and 168 h, respectively, and by 38% in leaves at 168 h.	[152]
**Endophytic plant growth-promoting bacterium**	*Paraburkholderia phytofirmans* PsJN	Four-week-old *Vitis vinifera* plantlets; root inoculation; roots and leaves analysed 1, 4, and 7 days after bacterization	Strongly upregulated PAL and stilbene synthase (STS) in roots from day 1 through day 7 and upregulated multiple flavonoid-pathway genes in leaves at days 4–7. PsJN also increased leaf anthocyanin accumulation and significantly altered the relative abundance of 32 metabolites in roots and 17 in leaves.	[153]
**Pathogenic bacterium**	*Pseudomonas syringae* pv. *Tomato* DC3000	*Arabidopsis thaliana* leaves during compatible infection	Caused detectable metabolic reprogramming within 8 h of infection, including changes in phenolic and indolic compounds, together with broader changes in amino acids, glucosinolates, sugars, and redox-related metabolites during disease establishment.	[154]
